# *Henryhalticusphilippinensis* gen. et sp. n., a minute halticine from the Philippines (Insecta, Heteroptera, Miridae, Orthotylinae)

**DOI:** 10.3897/zookeys.796.21240

**Published:** 2018-11-15

**Authors:** Gerasimos Cassis

**Affiliations:** 1 Evolution & Ecology Research Centre, School of Biological, Earth and Environmental Sciences, Sydney 2052 Australia School of Biological, Earth and Environmental Sciences Sydney Australia

**Keywords:** Heteroptera, Miridae, taxonomy, new taxon, Philippines

## Abstract

*Henryhalticusphilippinensis***gen. et sp. n.** is described from a single location in the Negros Oriental Province of the Philippines. The male and female genitalia are described and illustrated. On the basis of the genitalic characters, external morphology, and size and color, the new species is erected as a monotypic genus.

## Introduction

Representatives of the hyperdiverse family Miridae are found in most regions and terrestrial ecosystems of the world (Cassis and Schuh 2012), and as with other species-rich families, they display an astonishing range of morphologies and feeding preferences (Wheeler 2001). The subfamilial classification is stabilized, with eight subfamilies routinely recognized, of which the Orthotylinae are the third most diverse, with six included tribes (Cassis and Schuh 2012). The tribe Halticini is now routinely considered an ingroup within the Orthotylinae, although Wagner (1973) previously regarded it as worthy of subfamilial ranking, but this has no significant contemporary support.

The present work involves the discovery of a minute halticine species from the Philippines. Specimens have been held in the American Museum of Natural History for a considerable time, and numerous colleagues have been uncertain about its suprageneric position and if it represents a new taxon. In this work I assign it to the Halticini based on genitalic and pretarsal characters, and recognize it as a new genus and new species, with commentary about its affinities.

This work is dedicated to Dr. Thomas J. Henry, whom I have known from the early 1980s. My memory is not precise but I do recall going on a fieldtrip to the Cascade Mountains with Tom, a trip organized by my Ph.D. supervisor, the late John D. Lattin. I also recall near the end of the trip a very large fir tree had fallen across a dirt road, and we had no other option but to retrace our tracks, adding many hours to our return journey. This gave us many hours to talk about the Miridae, a journey that we share with few other entomologists. Tom has worked with the previous miridology greats, including his close friend, the late Jose Carvalho. In the ensuing years he has become one of the mirid greats himself, and it is an honor to name a new genus after him. I wish him well in his taxonomic and personal exploits in the years to come.

## Materials and methods

Specimens were borrowed from the American Museum of Natural History (**AMNH**). Two pairs of paratypes are to be housed at the University of New South Wales (**UNSW**). The specimens were digitized in the Plant Bug Inventory database (https://research.amnh.org/pbi/locality/).

Male and female genitalia were macerated in 5% KOH, rinsed in distilled water, and dissected and examined in glycerol. The genitalia were illustrated using a camera lucida attached to a Leica DMB compound microscope. External characters were examined and measured using a Leica 205C automated stereomicroscope and Leica digital software. External characters were also documented with a Hitachi Desktop TM3000 scanning electron microscope.

## Taxonomy

### Henryhalticus gen. n.

Taxon classificationAnimaliaHemipteraMiridae

http://zoobank.org/813CB8C6-AC79-4BB9-A895-B210BE065EA9

**Figure 1.**
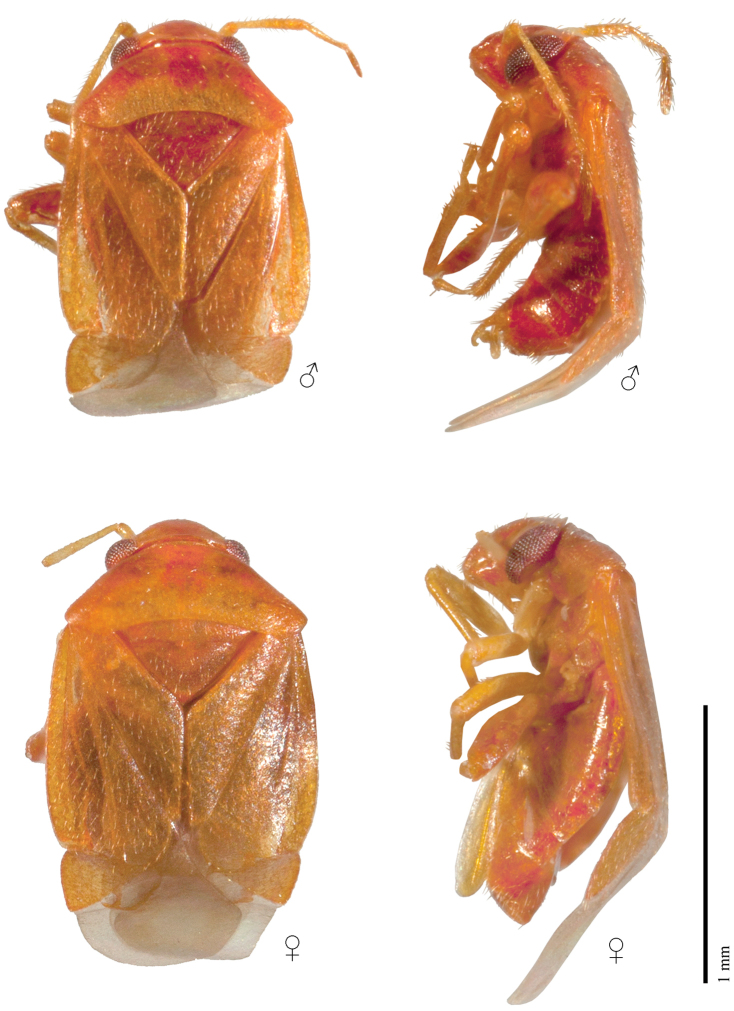
Male and female representatives of *Henryhalticusphilippinensis* gen. et sp. n.; dorsal and lateral views.

**Figure 2.**
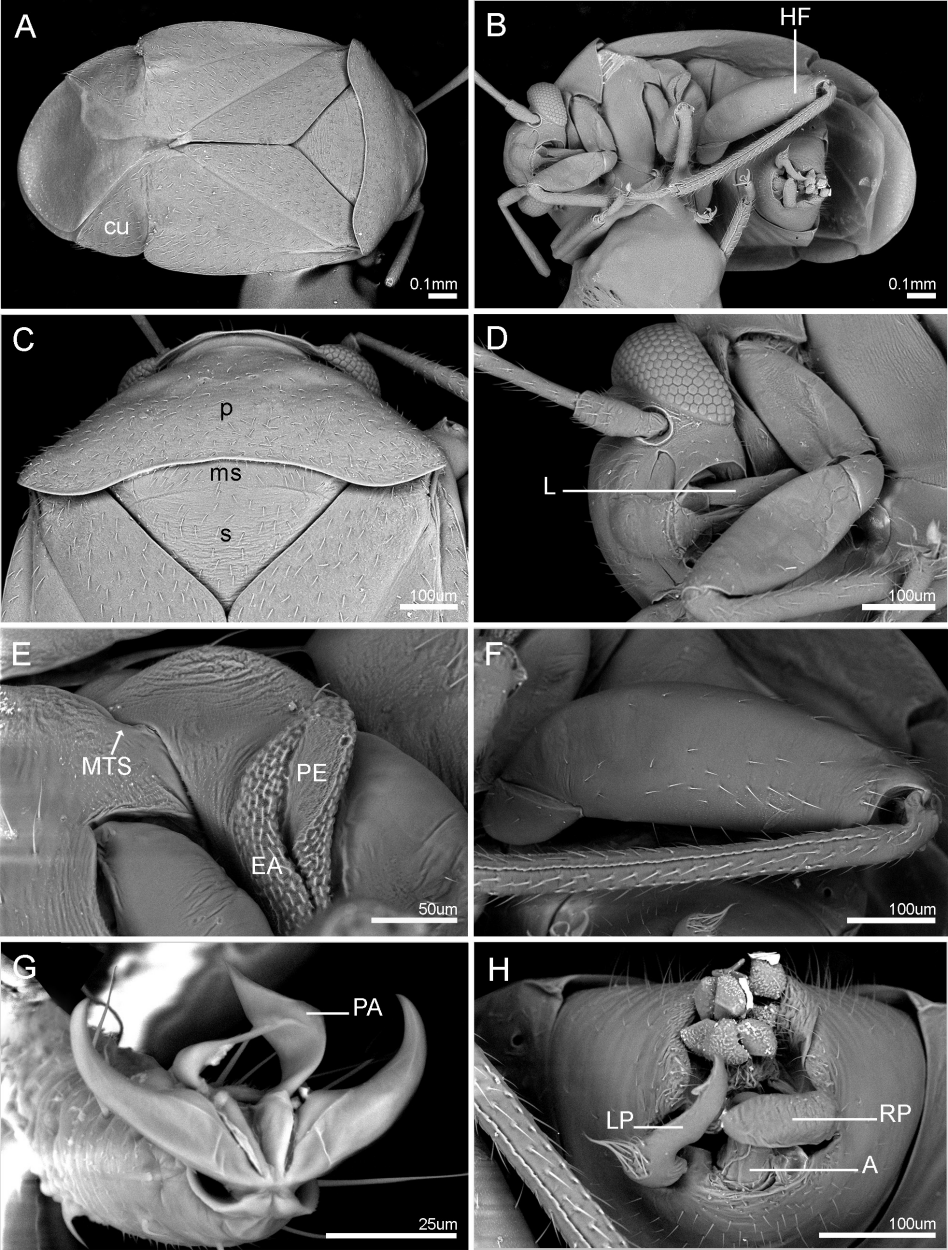
Scanning electron micrographs of key characters of *Henryhalticusphilippinensis* gen. et sp. n. **A** Dorsal view of body **B** Ventral view of body **C** Pronotum and scutellum **D** Lateroventral view of head and thorax **E** Pterothoracic pleura, incl. external efferent system of metathoracic glands **F** Metafemur and metatibia **G** Pretarsus, dorsal view **H** Genital opening of pygophore. Abbreviations: A = aedeagus; cu = cuneus; EA = evaporative area; HF = hind femur; L = labium; LP = left paramere; ms = mesoscutum; MTS = metathoracic spiracle; PA = parempodia; PE = peritreme; p = pronotum; RP = right paramere; s = scutellum.

**Figure 3.**
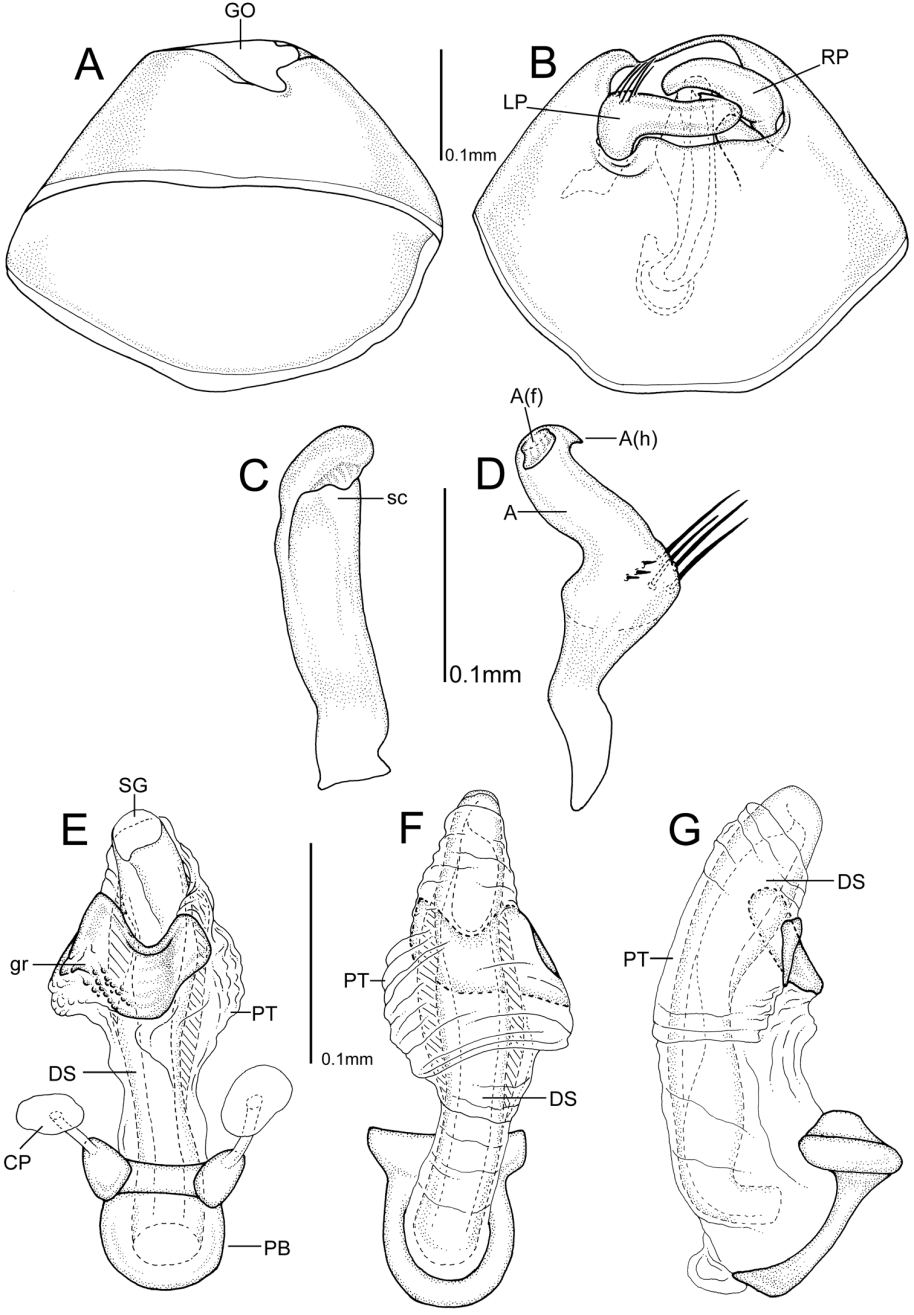
Male genitalia of *Henryhalticusphilippinensis* gen. et sp. n.

**Figure 4.**
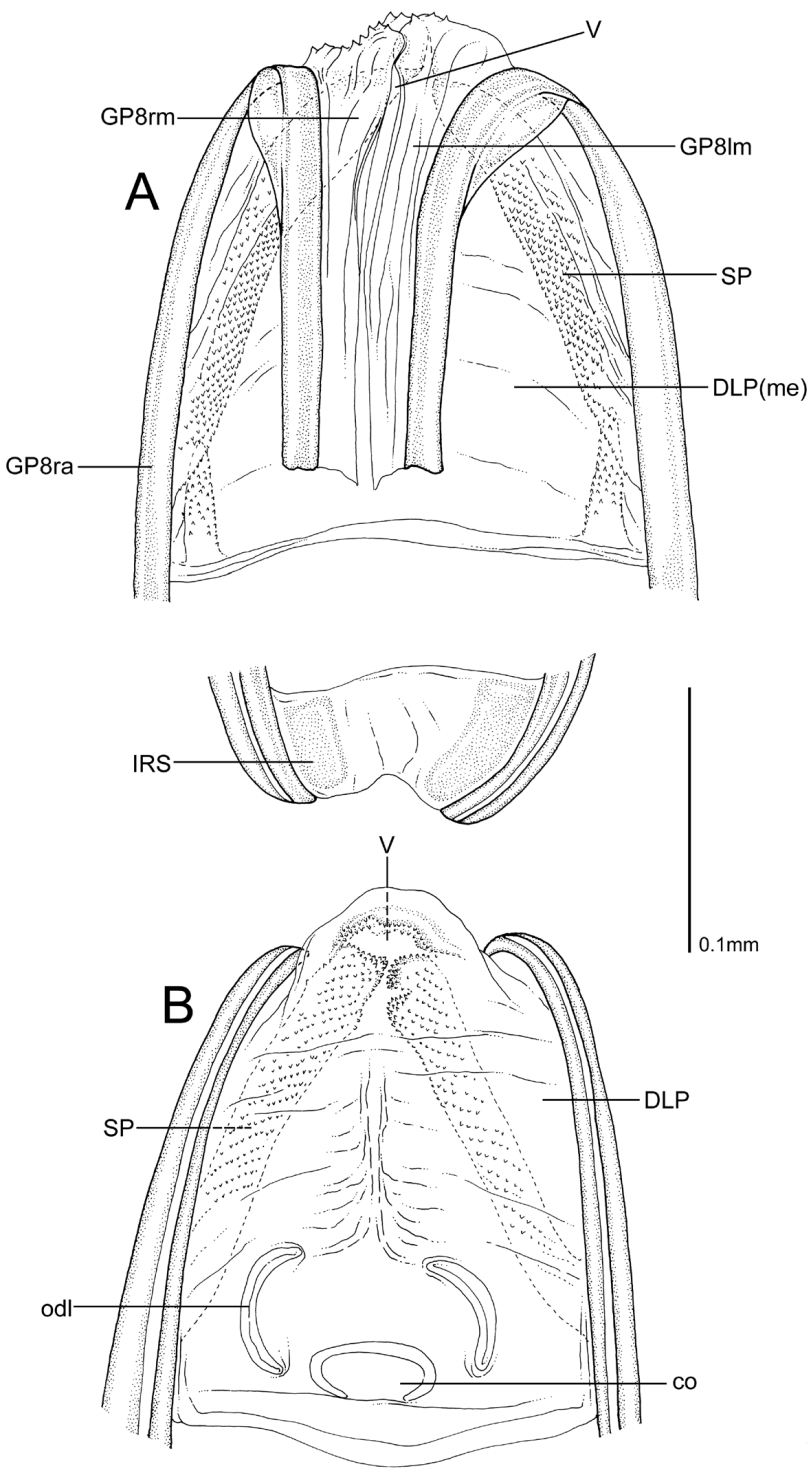
Female genitalia of *Henryhalticusphilippinensis* gen. et sp. n. **A** Ventral view of external female genitalia, including posterior wall of bursa copulatrix **B** Dorsal view of external female genitalia. Abbreviations: co = common oviduct; DLP(me) = dorsal labiate plate, mesial surface; GP8lm = gonapophyses 8 basal left membrane; GP8rm = gonapophyses 8 basal right membrane; IRS = inter-ramal sclerite; odl = lateral oviduct; SP = spiculate area of mesial surface of dorsal labiate plate; V = vestibulum.

Figures 1 , 2 , 3 , 4

#### Type species.

*Henryhalticusphilippinensis* sp. n., by original designation.

#### Diagnosis.

*Henryhalticus* is recognized by the following combination of characters: body minute, oval (Figs 1, 2A,B); posterior margin of head weakly carinate (Figure 2C); antennae inserted in front of and dorsad of ventral margin of eyes (Figure 2D); first antennal segment short (Figure 2D); labium very short, reaching only procoxae; costal fracture deep (Figs 1, 2A); evaporative area restricted to posterior margin of metepisternum, not reaching mesepimeron (Figure 2E); metafemora greatly enlarged (Fig. 2B, F); parameres overlapping (Figs 2H, 3B); endosoma without sclerotization (Fig. 3E–G); secondary gonopore large, apical, extending to apex of phallotheca at rest (Fig. 3E–G).

#### Description.

STRUCTURE. Body minute, oval, dorsal surface weakly convex, cuneus strongly deflexed (Figs 1, 2A, B). Head strongly dorsoventrally oriented, face strongly convex (Figs 1, 2A, B, C); vertex partly carinate (Figure 2C); eyes contiguous with anterior margin of pronotum (Fig. 2A, B, C). Antennae short, inserted anterior to eyes, and dorsad to ventral margin of eyes (Figure 2D); AI shorter than interocular distance; AII shorter than anterior margin of pronotum. Labium thick, very short, reaching only procoxae (Figure 2D). Pronotum large, subtrapezoidal; without collar; lateral margins linear; humeral angles broadly rounded; posterior margin deeply excavated (Figs 1, 2C). Scutellum large, broad, with mesoscutellum broadly exposed (Figs 1, 2C). Hemelytra oval, with costal margins broadly rounded; clavus large; embolium thickened; costal fracture deeply incised; cuneus transverse, lateral margins rounded; two membrane cells present, large vein rounded (Figs 1, 2A). Metathoracic gland efferent system well developed, arcuate, with peritreme reaching dorsal margin of metepisternum; peritreme tongue-like, reaching dorsal margin of evaporative area; evaporative area not reaching mesepimeron; metathoracic spiracle weakly exposed, without evaporative bodies (Figure 2E). Profemora and mesofemora small, metafemora greatly enlarged (Fig. 2B, F); parempodia lyre-shaped (Figure 2G). Male parameres overlapping (Figs 2H, 3B); aedeagus without sclerotization, secondary gonopore apical (Fig. 2E–G). Female posterior wall without inter-ramal lobes (Figure 3A).

See species description for coloration, texture, vestiture, and fine details of genitalia.

#### Remarks.

*Henryhalticus* is unlike other halticines in color, size, and shape. This is one of the smallest mirids described, with both sexes <2 mm in length. This genus keys to the Australian genus *Goodeniaphila* Tatarnic, in Tatarnic and Cassis’ (2012) global conspectus of the tribe Halticini. As with this latter genus, *Henryhalticus* lacks inter-ramal lobes on the posterior wall of the bursa copulatrix, but differs by possessing sclerotized rings on the dorsal labiate plates of the internal female genitalia. These two genera share a similar oval body, but *Henryhalticus* lacks any punctation or rugosity on the body, whereas *Goodeniaphila* has a rugose pronotum. The parempodia of these two genera are similar; however, *Henryhalticus* lacks tarsal pulvilli, in contrast to the former genus, which has large pulvilli. The male aedeagus is the most distinctive fine-scale difference between these two genera, with *Goodeniaphila* having multiple large endosomal spicules, whereas *Henryhalticus* lacks any endosomal sclerotization.

The aedeagus of *Henryhalticus* is most like that of *Halticus* Hahn. Both genera lack endosomal sclerotization, the posterior margin of the head is contiguous with the pronotum, and the posterior margin of the vertex is carinate. *Henryhalticus*, however, lacks sclerotized rings and tarsal pulvilli, and the efferent system of metathoracic glands is less well developed. In addition, the pronotum is more rounded posteriorly in *Halticus* and the mesoscutum is not as exposed in *Henryhalticus*.

### Henryhalticus philippinensis sp. n.

Taxon classificationAnimaliaHemipteraMiridae

http://zoobank.org/B609A85C-A09F-41C2-B51C-ECD56795A9AE

Figures 1 , 2 , 3 , 4

#### Material examined.

**Holotype**: PHILIPPINES: Negros Oriental: Camp Lookout, Dumaguete, Negros Island, 9.294°N 123.218°E, 396 m, 15 Feb 1961 - 15 Apr 1961, T. Schneirla & A. Reyes, ♂ (UNSW_ENT 00029095) (AMNH). **Paratypes**: PHILIPPINES: same locality as holotype; 15 Feb 1961 - 15 Apr 1961, T. Schneirla & A. Reyes, 15♂♂ (UNSW_ENT 00029096-UNSW_ENT 00029110) (AMNH); 06 Mar 1961, T. Schneirla & A. Reyes, 1♂ (UNSW_ENT 00029126) (AMNH); 21 Apr 1961, T. Schneirla & A. Reyes, 4♂♂ (UNSW_ENT 00029132-UNSW_ENT 00029135) (AMNH); 02 May 1961, T. Schneirla & A. Reyes, 2♂♂ (UNSW_ENT 00029118, UNSW_ENT 00029119), 3♀♀ (UNSW_ENT 00029120-UNSW_ENT 00029122) (AMNH); 03 May 1961, T. Schneirla & A. Reyes, 2♂♂ (UNSW_ENT 00029124, UNSW_ENT 00029125) (AMNH); 18 May 1961, T. Schneirla & A. Reyes, 1♂ (UNSW_ENT 00029131) (AMNH); 20 May 1961, T. Schneirla & A. Reyes, 22♂♂ (UNSW_ENT 00029136-UNSW_ENT 00029157), 6♀♀ (UNSW_ENT 00029158-UNSW_ENT 00029163) (AMNH); 21 May 1961, T. Schneirla & A. Reyes, 2♂♂ (UNSW_ENT 00029127, UNSW_ENT 00029128) (AMNH); 22 May 1961, T. Schneirla & A. Reyes, 2♂♂ (UNSW_ENT 00029129, UNSW_ENT 00029130) (AMNH); 24 May 1961, T. Schneirla & A. Reyes, 7♂♂ (UNSW_ENT 00029172-UNSW_ENT 00029178) (AMNH); 25 May 1961, T. Schneirla & A. Reyes, 10♂♂ (UNSW_ENT 00029179-UNSW_ENT 00029188), 3♀♀ (UNSW_ENT 00029190-UNSW_ENT 00029192) (AMNH); 26 May 1961, T. Schneirla & A. Reyes, 6♂♂ (UNSW_ENT 00029194-UNSW_ENT 00029199), 5♀♀ (UNSW_ENT 00029193-UNSW_ENT 00029203) (AMNH); 28 May 1961, T. Schneirla & A. Reyes, 6♂♂ (UNSW_ENT 00029164-UNSW_ENT 00029169), 2♀♀ (UNSW_ENT 00029170, UNSW_ENT 00029171) (AMNH).

#### Diagnosis.

*Henryhalticusphilippinensis* is recognized by the following combination of characters: body oval; minute, <2 mm in length; orange (Figure 1); body with moderately dense distribution of decumbent simple setae (Figs 1, 2A, C); genital opening of pygophore terminal, oval (Figs 2H, 3A, B); left paramere V-shaped, with apex recurved, plus apical flange (Figure 3C); right paramere upright, spoonlike apically (Figure 3D); ovipositor greatly elongate (Figure 1); gonapophyses 8 basally membraneous, nearly symmetrical (Figure 4A); posterior wall simple, membraneous, laterally spiculate, without discrete inter-ramal lobes (Figure 4B).

#### Description.

***Coloration.*** Body and appendages orange, with anterior lobe of pronotum, femora, clypeus and lateral regions of head, thoracic pleura and abdominal venter partly reddish orange; exocorium partly hyaline; hemelytral membrane smoky (Figure 1).

***Vestiture.*** Body with moderately dense distribution of decumbent hairlike setae; setae more erect on antennae, legs, and abdominal venter (Figs 1, 2A, B).

***Texture.*** Impunctate, shiny (Figs 1, 2A, B).

***Structure.*** As in generic description.

MALE GENITALIA. Pygophore conical (Figure 3A, B); genital opening terminal, large, suboval (Figs 2H, 3A, B). Parameres interlocking (Figs 2H, 3B); right paramere upright, distally spoon-shaped (Figure 3C); left paramere v-shaped, sensory lobe weakly developed with a few stiff bristlelike setae, apophysis short, with apex recurved, and with a flange-like process (Figure 3D). Aedeagus simple; phallotheca simple, mostly membraneous, with mediodorsal region weakly denticulate; ductus seminis broad, weakly ribbed; secondary gonopore broad, opening apically (Fig. 3E–G).

FEMALE GENITALIA. Ovipositor greatly elongate, almost reaching thoracic-abdominal boundary (Figure 1); membraneous regions of gonapophyses 8 nearly symmetrical (Figure 4A); vestibulum simple, with membraneous apex weakly denticulate (Figure 4A); mesial region of dorsal labial plate narrowly spiculate (Figure 4B); posterior wall of bursa copulatrix simple, membraneous, with lateral regions weakly spiculate (Figure 4A).

#### Measurements.

See Table 1.

**Table 1.** Measurement of key characters of *Henryhalticusphilippinensis*. All measurements given in millimeters. Mean, standard deviation, range, minimum, and maximum values given for each species. Abbreviations: CunClyp = maximum length between apex of clypeus and tip of cuneus, Pron = pronotum, Scut = scutellum, InterOc = Interocular distance, AntSegI–IV = antennal segment I–IV.

		Length						Width			InterOc	AntSegI	AntSegII	AntSegIII	AntSegIV
		Body	Cun Clyp	Head	Pron	Scut	Cun	Head	Pron	Scut					
M (n=5)	Mean	1.607	1.090	0.152	0.215	0.184	0.294	0.428	0.690	0.299	0.256	0.082	0.346	0.196	0.177
	SD	0.104	0.061	0.033	0.017	0.014	0.020	0.023	0.035	0.016	0.007	0.010	0.025	0.003	0.004
	Range	0.287	0.158	0.091	0.042	0.042	0.054	0.069	0.094	0.048	0.017	0.031	0.079	0.007	0.009
	Min	1.493	1.029	0.101	0.196	0.163	0.272	0.388	0.624	0.278	0.249	0.067	0.305	0.193	0.173
	Max	1.781	1.187	0.192	0.238	0.205	0.326	0.457	0.718	0.326	0.266	0.098	0.384	0.199	0.181
F (n=5)	Mean	1.774	1.207	0.161	0.222	0.212	0.289	0.493	0.860	0.374	0.263	0.114	0.311	0.085	0.164
	SD	0.131	0.056	0.045	0.031	0.021	0.052	0.007	0.015	0.019	0.021	0.021	0.170	0.000	0.000
	Range	0.357	0.147	0.119	0.088	0.057	0.117	0.021	0.046	0.051	0.063	0.048	0.341	0.000	0.000
	Min	1.525	1.147	0.073	0.194	0.181	0.235	0.482	0.843	0.351	0.232	0.095	0.140	0.085	0.164
	Max	1.882	1.294	0.192	0.281	0.238	0.352	0.502	0.889	0.401	0.294	0.143	0.481	0.085	0.164

#### Distribution.

Known only from the type locality on Negros Island, in the Philippines, between February 15 and May 28, 1961.

**Remarks.** See generic remarks.

## Supplementary Material

XML Treatment for Henryhalticus

XML Treatment for Henryhalticus philippinensis

## References

[B1] Cassis G Schuh RT (2012) Systematics, biodiversity, biogeography and host plant associations of the Miridae (Insecta: Hemiptera: Heteroptera). Annual Review of Entomology 57: 377–404. 10.1146/annurev-ento-121510-133533 22149267

[B2] Tatarnic NT Cassis G (2012) The Halticini of the World (Insecta: Heteroptera: Miridae: Orthotylinae): generic reclassification, phylogeny and host plant associations. Zoological Journal of the Linnean Society 164: 558–658.

[B3] Wagner E (1973) Die Miridae Hahn, 1831, des Mittelmeerraumes und der Makaronesischen Inseln (Hemiptera, Heteroptera). Entomologishe Abhandlungen 39: 1–423.

[B4] Wheeler Jr AG (2001) Biology of the Plant Bugs (Hemiptera: Miridae). Pests, Predators, Opportunists. Cornell University Press, Ithaca, 507 pp.

